# Texture Variations Suppress Suprathreshold Brightness and Colour Variations

**DOI:** 10.1371/journal.pone.0114803

**Published:** 2014-12-12

**Authors:** Andrew J. Schofield, Frederick A. A. Kingdom

**Affiliations:** 1 School of Psychology, University of Birmingham, Edgbaston, Birmingham, United Kingdom; 2 McGill Vision Research, McGill University, Department of Ophthalmology, McGill University, Montreal, Canada; University of Melbourne, Australia

## Abstract

Discriminating material changes from illumination changes is a key function of early vision. Luminance cues are ambiguous in this regard, but can be disambiguated by co-incident changes in colour and texture. Thus, colour and texture are likely to be given greater prominence than luminance for object segmentation, and better segmentation should in turn produce stronger grouping. We sought to measure the relative strengths of combined luminance, colour and texture contrast using a suprathreshhold, psychophysical grouping task. Stimuli comprised diagonal grids of circular patches bordered by a thin black line and contained combinations of luminance decrements with either violet, red, or texture increments. There were two tasks. In the Separate task the different cues were presented separately in a two-interval design, and participants indicated which interval contained the stronger orientation structure. In the Combined task the cues were combined to produce competing orientation structure in a single image. Participants had to indicate which orientation, and therefore which cue was dominant. Thus we established the relative grouping strength of each cue pair presented separately, and compared this to their relative grouping strength when combined. In this way we observed suprathreshold interactions between cues and were able to assess cue dominance at ecologically relevant signal levels. Participants required significantly more luminance and colour compared to texture contrast in the Combined compared to Separate conditions (contrast ratios differed by about 0.1 log units), showing that suprathreshold texture dominates colour and luminance when the different cues are presented in combination.

## Introduction

Demonstrations such as the Adelson checker-shadow illusion [1] show that humans are good at separating spatial changes in illumination from spatial changes in reflectance – a process termed ‘layer decomposition’ – and thus identifying, for example, the colours of surfaces irrespective of their illumination. Kingdom [2] provides an extensive review of the layer decomposition process, describing how it helps in the detection of object boundaries and in the identification of material properties and the shapes of objects (for example, via shape from shading). However, luminance changes (or ‘contrasts’ as often termed here) are ambiguous in this regard, since they can arise from both material and illumination changes. In order to determine whether a given luminance change results from a material or illumination change our visual system must combine it with other information, for example the fact that shadows tend to have soft edges [3]. Other useful information comes from the relationships between luminance changes and other, nearby, luminance [4], [5], colour [6]–[8], and texture [9]–[11] changes, as well as from the statistics of the image as a whole [12]. This knowledge is most likely acquired through experience, though some of it might be innate.

Before proceeding, a quick note on terminology. We use the term ‘colour’ to refer to the chromatic properties of a stimulus, ‘luminance’ for light intensity, and ‘texture’ for a surface with a dense array of luminance markings. Colour and texture changes tend to be primarily material in origin, and hence less ambiguous than luminance changes [2]. More specifically it is the *relationship* between changes in colour and luminance [7], [13], [14] and changes in texture and luminance [11], [15] that promotes the discriminability of material from illumination changes. Such supra-threshold interactions occur despite the separability at detection threshold between colour and luminance contrasts [16]–[23], and between texture and luminance contrasts [24], [25].

Although changes in colour do arise from illumination (e.g. a red sunset against a blue sky; a blue shadow against a white surround), in natural scenes such changes tend to be confined to the short wavelength (blue-yellow or violet-lime) colour channel in primate vision [26]. Thus colour contrasts (particularly red-green) are a reliable cue to material changes and hence object segmentation. Colour contrasts also influence eye movements more than luminance contrasts in a natural visual search task even when the target is achromatic [27]. We might therefore expect changes in colour to dominate changes in luminance in grouping tasks, as found by Kingdom, Bell, Gheoghiu, & Malkoc [28]. Similarly we might expect some types of colour variation to dominate over others. For example, there is some evidence that red-cyan variations, which uniquely stimulate one of the two postreceptoral colour channels (see below) dominate over violet-lime variations, which stimulate the other colour channel [29]. It may not be merely coincidental that of the two types of colour variation, the red-cyan variations tend to be least contaminated by shadows and shading [26], [30], [31].

Like colour, some texture variations are linked to illumination changes. For example, on a curved, shaded, textured surface changes in the local orientation of the markings may co-vary with the degree of shading. However, many texture variations in natural scenes are uncorrelated with changes in illumination. Some visual textures of course arise from variations in illumination. For example, when a surface is physically rough, small shadows cast by local peaks will give rise to visual texture, and the contrast of such textures will vary with the illumination, being strongest for strong, oblique illuminants. However even these textures will typically vary independently from coarser-scale illumination variations such as shadows cast from other objects. Moreover, physically smooth surfaces are often textured as a result of localised changes in reflectance, and these too will vary independently from illumination. If such textured surfaces are matte, changes in illumination are directly linked to changes in the luminance of individual texture elements, and in the difference between light and dark elements such that their contrast remains constant [11]. Conversely, a change in the contrast of a patterned texture is a cue to a material change both in its own right and when paired with luminance contrast [11], [15]. Thus, as with colour, modulations of the local luminance contrast of a texture (texture contrast) both cue material changes, and disambiguate the role of luminance changes. Therefore we might expect texture contrast to dominate luminance contrast in a suprathreshold task such as that used by Kingdom et al. [28]. On the other hand, Amano & Foster [27] found that local luminance contrast (of which our texture cue might be considered a sub set) did not influence eye movements in a visual search task as strongly as luminance or colour. This might suggest that texture contrast will be less potent as a grouping/segmentation cue than either colour or luminance.

**Figure 1:**
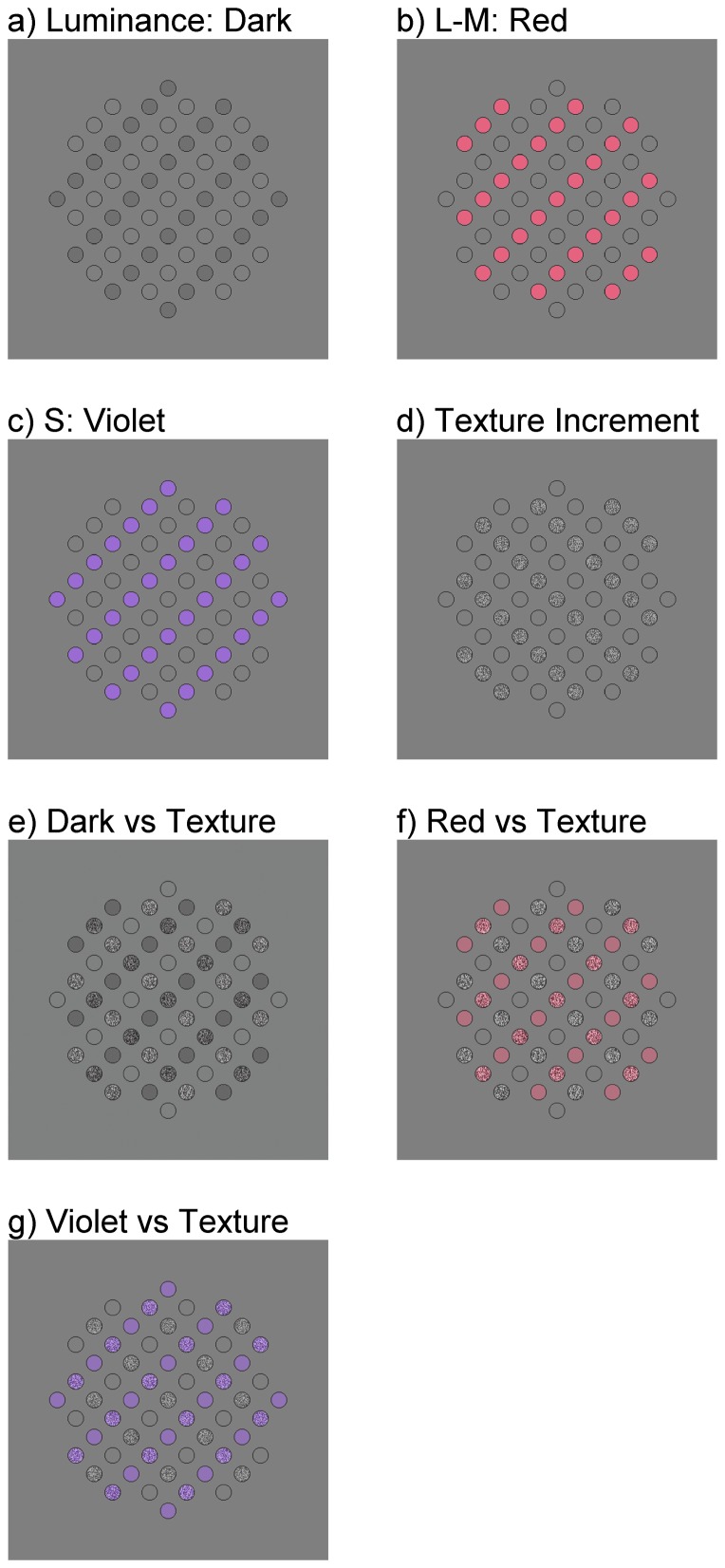
Example stimuli. Panels a-d Separate condition: a) Luminance decrements (dark) are arranged on alternate diagonal rows of a 2D lattice of circles. b) L-M colour axis increments (red). c) S colour axis increments (violet). d) Texture increments. Panels e-g Combined condition: e) Luminance decrements on one axis of the diagonal lattice are paired with texture contrast increments on the orthogonal diagonal. f) L-M colour axis increments (red) paired with texture contract increments. g) S colour axis increments (violet) paired with texture contrast. Cue contrasts have been exaggerated for publication.

Here we test for supra-threshold interactions between texture contrast, luminance contrast and colour contrast using a method devised by Kingdom et al. [28] based on a stimulus originally designed by Regan & Mollon [32]. This method measures the relative saliencies of two cues, both when the cues are presented separately (Separate condition) and when combined (Combined condition). Examples of the stimuli used in the Separate conditions are shown in Fig. 1a-d, and for the Combined conditions in Fig. 1e-g. Suprathreshold interactions between the cues are evidenced by a shift in the point-of-subjective-equality (PSE) of the two cues when going from the Separate to Combined condition. The Separate condition provides the necessary baseline in order to establish whether the results from the Combined condition implicate a genuine interaction between the two cues rather than simply a difference in their saliencies. For example, if texture contrast is a cue to material boundaries then, like colour [28], we might expect it to dominate luminance contrast when the two cues are combined. This would be evidenced by a shift in the PSE *towards luminance contrast* when going from the Separate to the Combined condition, as more luminance contrast would be needed to balance texture contrast when the two cues were combined.

## Method

### Procedure

There were two main conditions, Separate and Combined. Each condition was tested in three cue comparisons: Texture vs. Dark, Texture vs. Red and Texture vs. Violet. Conditions and comparisons were blocked by session. In the Separate condition the two components were each presented for 500 ms in separate stimuli (Fig. 1a-d) with a 500 ms inter-stimulus interval. Observers pressed a key to indicate the stimulus they perceived as containing the more salient orientation structure and were told that “more salient” was synonymous with “more pronounced oblique orientations”. Trials were initiated by the previous response, with a 500 ms delay before the first stimulus. Eight ratios of the contrasts of the two components were presented in random order, with 20 trials per ratio and 160 trials per session. The contrast of each component was selected from 8 logarithmically-spaced values chosen to span the full range of performance for each participant as shown in S1 Dataset. Test contrasts were indexed a1 to a8 for one component and b1 to b8 for the other and were paired as follows: a1 & b8; a2 & b7; a3 & b6; a4 & b5; a5 & b4; a6 & b3; a7 & b2; a8 & b1. We did not combine the colour and luminance conditions as these have already been tested using an almost identical method [28] but we compare our results with those of the previous study in the discussion.

In the Combined condition the two components were presented on opposite oblique axes in a single stimulus (Fig. 1e-g). On each trial the stimulus was presented for 500 ms and a key press indicated the orientation, left- or right-oblique, that was more salient. The conditions were otherwise identical.

### Stimuli

The stimuli were generated on a VISAGE graphics system (Cambridge Research Systems, CRS, Rochester, UK) and displayed on a Sony Trinitron F500 CRT monitor (Sony, Tokyo, Japan). The red, green and blue outputs of the monitor were gamma-corrected after calibration with a CRS Optical photometer. The CIE coordinates of the monitor's phosphors were red: *x* = 0.624, *y* = 0.341; green: *x* = 0.293, *y* = 0.609; and blue: *x* = 0.148, *y* = 0.075.

The component patterns were generated on separate video pages, along with their own calibration look-up-tables (LUTs). During stimulus presentation the two video pages (and corresponding LUTs) were alternated at the monitor frame rate of 120 Hz; overall refresh rate  =  60 Hz. For the Separated condition the component patterns alternated with a mid-grey screen, whereas in the Combined condition the component patterns alternated with each other ensuring that there were no artifactual within-image interactions between the components. Frame alternation halves the effective contrast of each component and we report the halved values below.

Stimuli comprised a grid (diameter 8.9 deg at the 110 cm viewing distance) of circles (diameter 0.383 deg) each ringed by a 1 pixel-wide black line. The black rings reduced the impression of transparency in the Combined condition and masked any chromatic aberrations. The separation between circles was 0.68 deg along the oblique axes and 0.96 deg along the cardinal axes. Cues were applied to alternate runs of circles on one or other of the oblique axes.

There were four cues: Violet, Red, Dark and Texture. The first three of these were defined along one pole of an axis in the DKL colour space [16] itself a modified version of the MacLeod-Boynton [33] colour space. The colour axes were defined in terms of long- (L), middle- (M) and short- (S) wavelength cone-contrasts as follows: 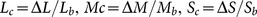 [34]–[37]. The denominator in each cone-contrast term refers to the cone excitation produced by the mid-grey background (CIE chromaticity *x* = 0.282 and *y* = 0.311; luminance 40 cd/m^2^). The numerators represent the difference in cone excitation between the circle colour and the background. Desired LMS cone excitations were converted to RGB phosphor intensities using the cone spectral sensitivity functions provided by Smith & Pokorny [38] and the spectral emission functions of the monitor phosphors as measured with a PR640 spectral radiometer (Photo Research, Chatsworth, CA).

The Violet, Red and Dark colours/luminances employed here uniquely stimulate one of three post-receptoral mechanisms [16], [34]–[37], [39]. These mechanisms combine cone contrasts as follows: 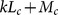 for the luminance mechanism (LUM), 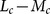 for the mechanism that differences L and M cone-contrasts (L-M), and 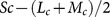 for the mechanism that differences S from the sum of L and M (S). The parameter *k* determines the relative weightings of the L and M inputs to the luminance mechanism, and was established separately for each observer by estimating their isoluminant point using the minimum perceived motion method (see [28] for details). We used the same method to ensure that the S axis stimuli introduced no luminance artefact. Table 1 shows the values of *k* and the Lum∶S ratio at isoluminance for the 7 observers.

**Table 1:** Isoluminance measures for all observers.

Observer	*k*	Lum∶S ratio
P1	1.71	.048
P2	0.99	.073
P3	1.39	.084
P4	1.12	.043
P5	1.18	.076
P6	1.7	.07
P7	0.93	.077

Full isolation of the three cardinal mechanisms was achieved using the following equations (after [13]):
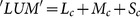
(Eqn.1a)

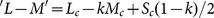
(Eqn.1b)

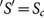
(Eqn.1c)


The measures of contrast for the Violet, Red and Dark colours/luminances were calculated as follows: for Dark, we used the contrast assigned to each of the three cones (i.e. 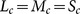); for Red, the difference between 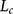 and 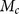; and for Violet, simply 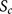.

For Texture stimuli we used a binary noise pattern in which each pixel was randomly allocated one of two luminance levels which were equal amplitudes from the background mid-grey value and thus added no low-frequency luminance signal. The two grey levels fell on the LUM axis of colour space and thus introduced no signal in the L-M or S mechanisms. The contrast of any cue is defined as amplitude/mean on the relevant dimension with amplitude referring to the light and dark luminance values relative to mean luminance in the case of Texture stimuli.

### Participants and Ethics Statement

The seven observers had normal or corrected-to-normal visual acuity and normal colour vision as assessed by the Ishihara plates. Except for author FK (P6) all were undergraduate volunteers at McGill University who were naïve as to the purpose of the experiment. Observers gave their written informed consent prior to participating in this study and were treated in accordance with the Declaration of Helsinki. The research protocol was approved by the McGill University Health Centre Research Ethics Office.

### Data analysis

Psychometric functions were fitted with the Logistic function
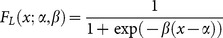
(Eqn 2)where *x* is the log (logarithm) ratio of component contrasts, *α* the PSE defined as the contrast ratio producing a proportion of 0.5 responses (that is, cues are perceived equally salient), and *β* the slope of the function. The fitting procedure, conducted with the Palamedes toolbox [40], used a maximum-likelihood criterion. PSE shifts 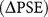 were calculated as the difference in PSE between the two Combined and Separate conditions.

## Results

**Figure 2:**
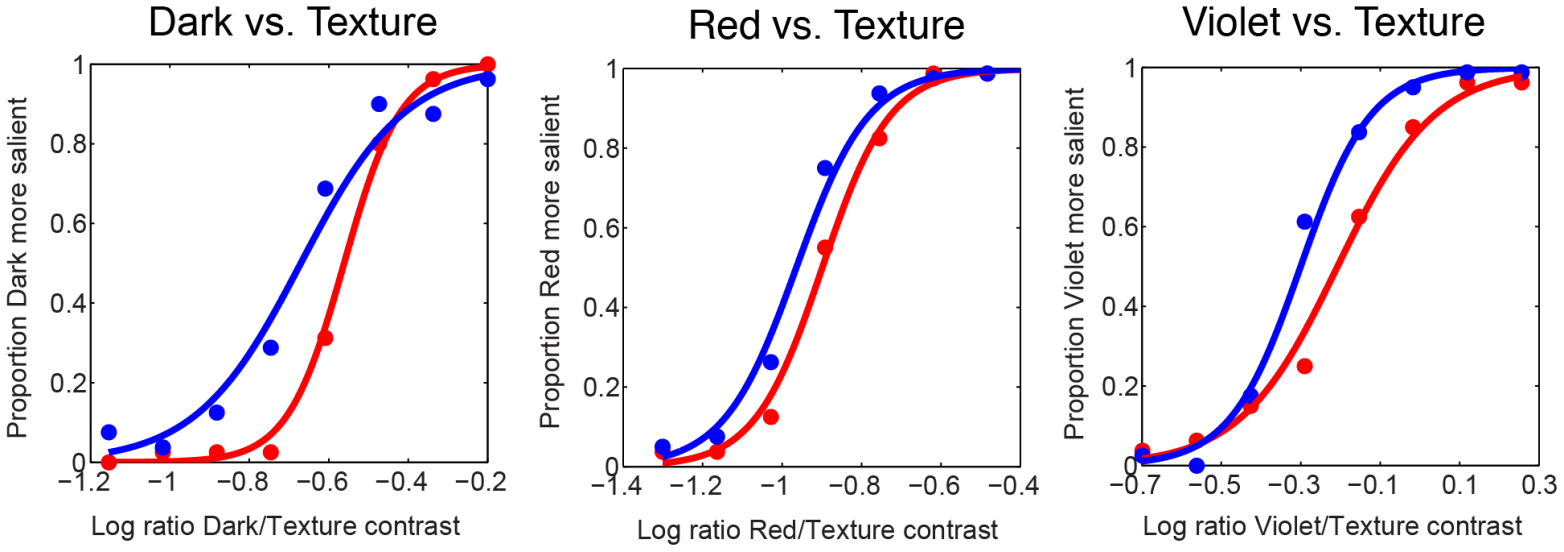
Example psychometric functions. Psychometric functions from observer P2, for all three cue combinations and the two presentation conditions: left: dark vs. texture, middle: red vs. texture, and right: violet vs. texture. Graphs show the proportion of trials on which the non-texture cue was judged to produce the stronger orientation structure as a function of the logarithm of the ratio of non-texture cue contrast to texture contrast. Blue symbols show data from the Separate condition; red Combined. Lines show best fit logistic functions – see main text.

Fig. 2 shows a complete set of psychometric functions for observer P2. Each graph plots the proportion of times the Dark, Red or Violet component was chosen as more salient than the Texture component as a function of the log contrast ratio of the two components. The blue and red lines are best fits for the Separate and Combined conditions, respectively. Since we are concerned only with differences in PSE between the two conditions, the absolute PSEs are not relevant, and in any case these will depend on the particular metric employed to measure the contrast of each cue. In all graphs, the Combined psychometric function falls to the right (positive shift) of the Separate psychometric function, indicating that less texture contrast relative to either luminance or colour contrast is needed to achieve the PSE in the Combined compared to Separate condition.

**Figure 3:**
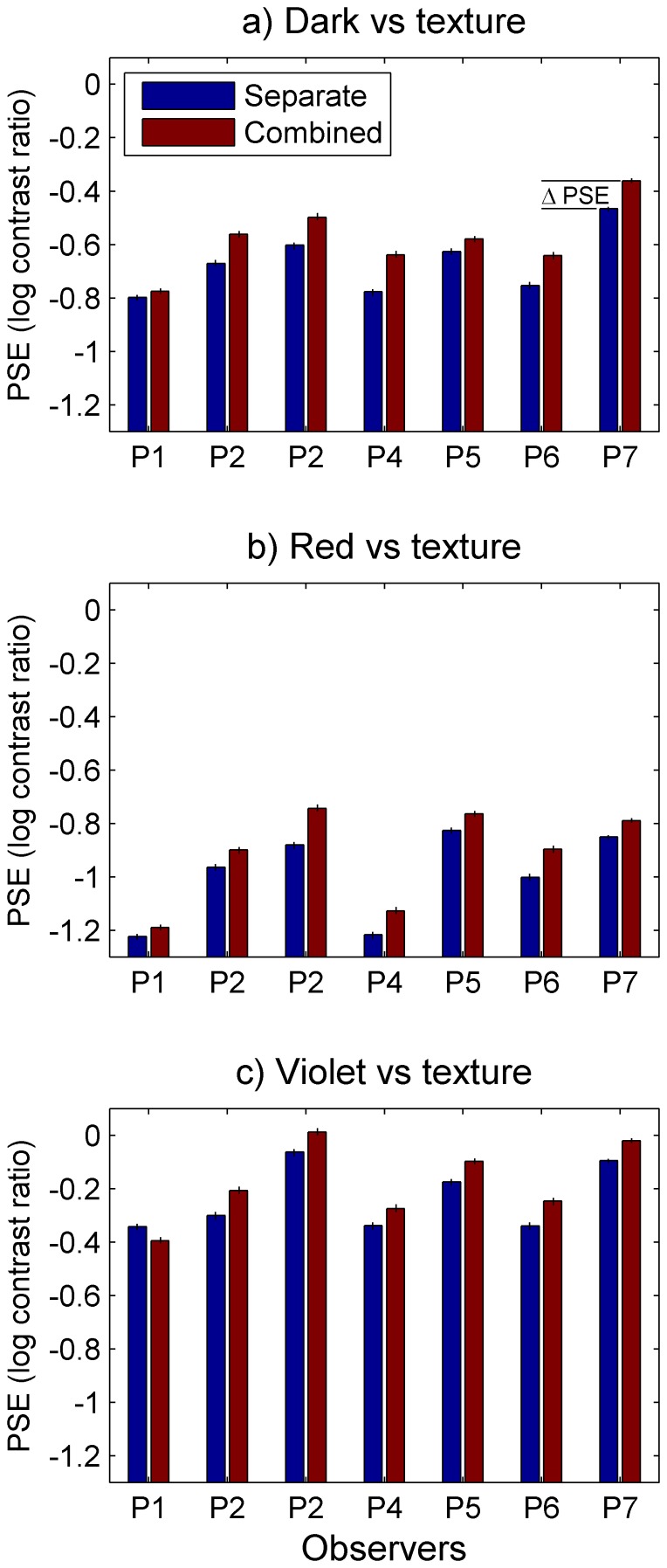
PSE estimates. PSE estimates are shown for seven observers for all three cue combinations and the two presentation conditions: a) dark vs. texture; b) red vs. texture; c) violet vs. texture. Blue bars show PSEs for the Separate condition; red Combined. Error bars show bootstrapped standard error estimates. Negative PSE's indicate that more texture contrast is required at the PSE but positive shifts in PSE between conditions indicate that less texture contrast is required in the Combined compared to Separate case.

Fig. 3 shows the PSE's for the Separate and Combined conditions for all observers. In all but one case (P1's Violet vs. Texture condition) the PSE shifts in the positive direction. Indicating that less texture contrast was required at the PSE in the Combined condition than the Separate condition. Paired sample t-tests show that the mean difference between PSEs (Mean PSE shifts, 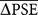) were positive and significant for all three comparisons (Dark vs. Texture, 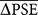 =  0.091, *SD* = 0.04, *t*(6) = 5.98, *p* = 0.001, Cohen's *d* = 2.26, *r* = 0.93; Red vs. Texture 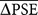 *= *0.079, *SD = *0.034, t(6) = 6.19, *p* = 0.001, *d* = 2.34, *r* = 0.93; and Violet vs. Texture, 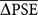 *= *0.06, *SD = *0.05, *t*(6) = 3.13, *p* = 0.02, *d* = 1.18, *r* = 0.79) indicating that texture contrast is dominant in the Combined conditions. The PSE shifts in log units given above correspond to changes in the contrast ratios as follows: 23% more luminance contrast, 19% more red contrast, and 14% more violet contrast were required in the Combined vs. Separate conditions. Shifts in PSE can be hard to interpret when the slope (*β*) of the psychometric function also changes between conditions. We therefore assessed the mean change in slope for each cue combination. Except for the Violet vs. Texture case these were not significant (Dark vs. Texture: 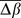 *= *−2.17, *SD* = 4.96, *t* = −1.16, *p* = 0.29; Red vs. Texture: 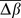 *= *−4.08, *SD* = 6, *t* = −1.8, *p* = 0.12; Violet vs. Texture 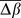 *= *−4.55, *SD* = 4.42, *t* = −2.72, *p*<0.035, *d* = −1.03, *r* = 0.74). Taken together these results suggest that PSE shifts were both significant and reliable for the Dark- and Red vs. Texture conditions whereas for the Violet vs. Texture case the PSE shift, while significant, is weaker and possibly confounded with changes in psychometric slope.

## Discussion

Humans are relatively insensitive to modulations of texture contrast. Typically, sensitivity to luminance gratings at 2 c/deg is 100 times higher than that for modulations of the contrast of random noise patterns (contrast modulations, CM, [24]) similar to our texture cue. Furthermore, whereas supra-threshold luminance modulations mask the detection of contrast modulated stimuli this masking is not reciprocated. Such findings suggest that texture contrast is at best a secondary cue to luminance contrast. However, other results have shown roughly equal suprathreshold interactions between the two cues (e.g. [25]). In addition, Schofield et al. [11], [15] have shown that texture contrast can have a profound effect on the appearance of luminance modulated stimuli changing their appearance from shaded undulations to strips of material change – the relationship between the cues being the critical factor. Similar effects have been shown for colour-luminance interactions [7], [8].

Given the ability of colour and texture to cue object boundaries, we might expect these cues to be relatively dominant in tasks that require an element of grouping or segmentation. Kingdom et al. [28] showed that colour tends to dominate luminance in an almost identical paradigm to that used here. Thus luminance is either more suppressed or less facilitated than colour when the two are presented in one stimulus. We have shown a similar result for texture vs. luminance contrast.

In our experiments luminance contrast ranged from 90–250 times contrast detection thresholds for 2 c/deg gratings. Texture contrast ranged from 3–10 times detection threshold for similar contrast modulated gratings [24]. Thus although the absolute contrast of our texture stimuli was higher than that for our luminance stimuli, in terms of threshold multiples the luminance cue was much the stronger at the measured PSEs. However the crucial point is that it is not their individual saliences that matter – these are factored out by the Separate condition – but the way they interact when combined.

We now compare our results to those of Kingdom et al. [28], [29]. On average, in the Dark vs. Texture comparison our observers required 23% more luminance contrast in the Combined vs. Separate condition. This compares to an average of 32% for the Luminance vs. Red-Cyan and for the Luminance vs. Violet-Lime comparisons tested by Kingdom et al. [28]. This might suggest that texture-contrast is less dominant than colour contrast. However, we found here that texture contrast dominated colour contrast: 19% for Red vs. Texture, 14% for Violet vs. Texture. Further, Kingdom et al. [29] found that red-cyan dominated violet-lime – though only by about 8% whereas here we find that texture contrast appears to be less dominant over violet contrast than it is over red contrast. It is clear that comparisons between any pair of cues cannot be inferred from their separate interactions with a third cue.

The above limitations notwithstanding, we suggest that the overall pattern of cue dominance (texture> colour> luminance) may reflect a hierarchy in which those cues that are more ambiguous with respect to material vs. illumination changes tend to be suppressed by those that are less ambiguous. Luminance is highly ambiguous in this regard, S-axis (violet-lime) colour variations less so, L-M (red-cyan) colour variations less still (hence Kingdom et al.'s, [29] result) and (we speculate somewhat) texture contrast the least ambiguous. In natural scenes luminance decrements and violet contrasts are more present in shadows than are red or texture contrasts [26], and at least for patterned (vs. rough) textures, texture changes seem only to arise from material changes [11]. If this account is correct then we would predict that abrupt variations in texture orientation – a clear cue to segmentation [41], [42] – will also dominate over luminance and colour contrast, although the synergy between shading and orientation cues to surface shape [9], [10] suggests that orientation changes might be less potent in this regard when consistent with a uniform texture on an undulating surface.

Finally the finding that colour dominates luminance [28] is supported by Amano & Foster's [27] finding that colour has greater influence over eye movements than luminance in a naturalistic visual search task. However, Amano & Foster [27] also found that local luminance contrast (measured as the standard deviation of pixel intensity values within small regions of an image; thus similar to our texture contrast cue) has a relatively weak influence over eye movements as compared to either luminance or colour. This is not consistent with our current result. It is possible that the naturally occurring local luminance contrast variations measured by Amano & Foster [27] were weak relative to the colour and luminance signals in their natural images whereas our texture contrast was deliberately matched in strength with to colour and luminance contrast by virtue of our use of the Separate cues condition as a base line.

In conclusion we have shown that – despite its relative weakness as a cue in absolute terms – texture contrast dominates luminance decrements and colour changes in a perceptual grouping task.

## Supporting Information

S1 Dataset Data including stimulus test ranges. This file contains raw data from all participants including the number of times each cue was considered more salient for each pair of test levels in all conditions, Isoluminance settings and the test levels used for each participant.
(XLS)
